# Governmental Measures in Switzerland Against Mass Bankruptcies During the Covid-19 Pandemic

**DOI:** 10.1007/s40804-023-00280-8

**Published:** 2023-03-16

**Authors:** Rodrigo Rodriguez, Jasmin Ulli

**Affiliations:** 1 Professor for Civil Procedure and Insolvency Law, University of Lucerne, Lucerne, Switzerland; 2 Head of the Supervisory Authority on Debt Collection and Insolvency, Swiss Federal Department of Justice and Police, Bern, Switzerland; 3 Research Assistant, University of Lucerne, Lucerne, Switzerland

**Keywords:** Pandemic, Insolvency, Stay, Loans, Debt enforcement, Moratorium, Over-indebtedness, Covid-19

## Abstract

This article examines the impact of the Covid-19 pandemic on debt enforcement and insolvency law in Switzerland. Despite the absence of strict lockdown measures, many sectors of the Swiss economy suffered significant losses. The government responded by introducing generous public support schemes to keep businesses afloat. The article focuses on the modifications made to Swiss law during the pandemic to avoid mass bankruptcies and facilitate restructurings. The government first introduced a general stay of proceedings, preventing debt collection but not affecting the underlying obligation to pay, and later the Covid-19 Ordinance on Insolvency Law, which provided relief to companies, especially SMEs, to implement the necessary restructuring measures. Some unsuccessful initiatives, such as a special moratorium introduced for SMEs, are also discussed. Finally, the article considers the limited take-up of some of the insolvency measures and discusses the possible consequences of the obligation to repay Covid-19 loans in the future. Overall, it provides a comprehensive overview of the impact of the pandemic on debt enforcement and insolvency law in Switzerland and the measures taken to mitigate its effects.

## Introduction

The Covid-19 pandemic hit Switzerland early and strongly in relation to the size of the population.^1^ Nevertheless, the restrictions imposed by the Swiss authorities on the economy and public life were modest in comparison with the neighboring countries. In particular, there was never an actual ‘lockdown’ or a curfew, people were allowed to leave the house at any time (but were strongly recommended not to do so), and the closing of schools was limited to a period of 2–3 months. Nevertheless, the impacts on trade in certain sectors (restaurants, tourism, transportation, entertainment) were massive. Generous public support schemes contributed significantly to keeping businesses and entrepreneurs afloat. These included generous loan schemes^2^ as well as covering the employment costs for temporarily laid-off employees^3^ (in exchange for maintaining the employment relationship).

Despite these measures, many sectors, especially those only indirectly hit by the pandemic (and with little or no access to public funds due to not being directly affected) suffered severe economic losses during the pandemic. In this article, we explore how pre-pandemic debt enforcement and insolvency law were modified in Switzerland during the Covid-19 pandemic in a bid to avoid mass filings and to facilitate restructurings. We report on the apparent failure of some of these initiatives, in particular a special moratorium introduced for SMEs, and suggest some lessons that can be learned from this. We do not consider in any detail the other forms of state support offered to businesses, such as Covid-19 loans and the ‘reduced employment’ scheme.^4^ We do, however, suggest that these other measures may help explain the limited take-up of some of the insolvency measures, and discuss some possible consequences of the obligation to repay Covid-19 loans in the years to come.

### Debt Collection and Bankruptcy Law in Switzerland in Short

Under Swiss law, a court judgment is not necessarily required to enforce private law claims. Enforcement proceedings can be initiated even for disputed claims. The relevant Act for debt enforcement as well as for insolvency proceedings is the Swiss Debt Enforcement and Bankruptcy Act **(‘**DEBA’) of 1889.^5^

### Measures Under the Swiss Debt Enforcement and Bankruptcy Act During the Covid-19 Pandemic

**Fig. 1:**
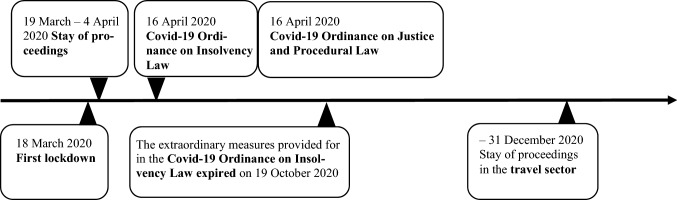
Measures taken under DEBA

Measures were taken under DEBA immediately after the first Covid-19 restrictions were put in place in March 2020. Interestingly, DEBA was one of the very few federal laws that had already (prior to the Covid-19 pandemic) made specific provision for pandemic situations. The first measure, taken under DEBA, was a general stay of proceedings. This was followed by a range of other insolvency measures designed to guard against mass bankruptcies, which are summarized in Fig. 1. Each of these measures is explored in more detail below.

## Stay of Proceedings in Accordance with Art. 62 DEBA

On 18 March 2020, the Swiss Federal Council decided on a collective stay of proceedings pursuant to Art. 62 DEBA for the entire territory of Switzerland.

The order was based on Art. 62 DEBA, which reads as follows:

In the event of an epidemic or a national disaster, or in times of war, the Federal Council or, with its consent, the cantonal government may grant a stay of proceedings for a certain region or part of the population.

The effect of this general stay (applicable to the whole of Switzerland and to all sectors) was that no enforcement proceedings could be initiated during the period in question (see below). This included even the notification of the beginning of enforcement proceedings. It was the first time in over 100 years that such a measure had been taken.

### Duration of the Stay of Proceedings

The stay of proceedings applied from 7.00 a.m. on 19 March 2020 until midnight on 4 April 2020, directly followed by the debt collection holidays pursuant to Art. 56(2) DEBA, so that the stay of proceedings lasted until 19 April 2020 (Art. 56 DEBA) and the extension of the deadline pursuant to Art. 63 DEBA until Wednesday 22 April 2020.^6^ During the stay, ‘except in attachment proceedings or in the case of measures that cannot be postponed for the preservation of assets, ... acts of debt collection may not be carried out’ (Art. 56 DEBA).

The stay of proceedings was not extended beyond this period of merely a few weeks. This is due to the fact that the stay of proceedings was ordered primarily for operational reasons (preparation for operation under pandemic conditions). It was seen as a measure that could considerably distress the economy, as the (negative) example of its last use during the First World War had demonstrated.^7^ Only for the travel industry did the Federal Council order a longer period. The latter stay was limited in scope (applying merely to claims against travel agencies for compensation as a consequence of cancelled travel arrangements) and ended on 31 December 2020.

### Effect of the Stay of Proceedings

The stay of proceedings is purely procedural, preventing debt collection, but having no influence on the underlying substantive obligation. To protect debtors not only temporarily from enforcement, but from the payment obligation itself, other instruments were needed (cf. remarks on the Covid-19 Ordinance on Insolvency Law below).^8^ Furthermore, according to Art. 63(1) DEBA, the stay of proceedings has no inhibiting effect on the statutes of limitation under debt collection law or on any contractual or statutory interests accruing. In short: the debt may temporarily not be enforced, but it is still due!

## Covid-19 Ordinance on Insolvency Law 2020

As early as at the beginning of the Covid-19 pandemic in Switzerland in March 2020, the Federal Council adopted the Covid-19 Ordinance on Insolvency Law (‘C19-InsolOrdinance’),^9^ in force as of 20 April 2020 on the basis of emergency law.^10^ The Ordinance was valid for 6 months, i.e., until 20 October 2020 (Art. 23(1) C19-InsolOrdinance).

With this Ordinance, the Federal Council wanted to relieve, by means of emergency legislation, the pressure on the boards of directors. The purpose of the Ordinance was to give companies (and especially SMEs) some breathing space to implement the necessary restructuring measures.^11^ In the field of insolvency, the Ordinance contained two main measures: an easing of the notification requirements for over-indebtedness in Art. 725 SCO (see Sect. 3.1) and a new restructuring proceeding (Sect. 3.2).

### Easing of Notification Obligations Pursuant to Art. 725 SCO

If a company is over-indebted—i.e., its liabilities are not covered by assets either at going concern or at liquidation values—the board of directors will ordinarily be obliged, under Art. 725(2) of the Swiss Code of Obligations (‘SCO’),^12^ to notify the bankruptcy court immediately. There are only three exceptions to this strict notification requirement:

1. Creditors have subordinated their claims to a sufficient extent;
2. There is a reasonable prospect of financial restructuring of the company within a reasonable, short period of time; or
3. The board of directors submits a (provisional) request for debt restructuring.

If none of these exceptions apply and the judge is not notified despite the existence of over-indebtedness, the responsible executive bodies face personal liability for damages resulting from a delayed declaration of bankruptcy (Art. 754(1) SCO).

To reduce the pressure on the board of directors, the Ordinance provided for a suspension of the notification obligations pursuant to Art. 725(2) SCO under certain circumstances. Thus, the board of directors (and secondarily also the auditors) could refrain from notifying the bankruptcy judge in case of over-indebtedness if the following conditions were cumulatively met (Art. 1(1) C19-InsolOrdinance): the company must not already have been over-indebted on 31 December 2019, and there must have been a prospect that the over-indebtedness could be eliminated by 31 December 2020.

The board of directors’ decision not to notify the court on the basis of the two conditions had to be documented in writing, e.g., in minutes of the meeting (Art. 1(2) C19-InsolOrdinance). In order to prove the positive future prognosis, corresponding documents such as interim balance sheets and liquidity plans had to be submitted; comprehensive restructuring and business plans as well as financing concepts and letters of intent could also be submitted.^13^

After the reintroduction of the duty to notify (in November 2020), there was, contrary to expectations, no sudden wave of over-indebtedness notifications (i.e., insolvencies). The reasons for this are unclear. In our opinion, other measures, such as the short-time work compensation^14^ or the Covid-19 loans granted,^15^ have probably had a massive influence on maintaining liquidity. There had also been a rise in solvent liquidations during the pandemic.

A further important measure to avoid (formal) over-indebtedness relates to the Covid-19 loans: under normal accounting provisions, the Covid-19 loans would weigh so heavily on the liabilities side of the balance sheets of many of the (formerly) credit-needy companies that they would result in formal over-indebtedness. Therefore, the Solidarity Guarantee Act provided that

[for] the calculation of the coverage of capital and reserves according to Article 725(1) SCO and for the calculation of over-indebtedness according to Article 725(2) SCO, loans guaranteed on the basis of Article 3 [C19-Solidarity Guarantee Ordinance] shall not be taken into account as debt capital.

Unlike the obligation to file under Art. 725 SCO, this measure (exclusion of Covid-19 loans from the calculus of over-indebtedness) has been retained^16^ and will apply until 31.12.2032 (by then, the Covid-19 loans would have to be repaid, see Sect. 5, *in finem*). This means that in the coming years many enterprises will ‘fly under the radar’ of the financial distress triggers of Art. 725 SCO despite having considerable debts that, if considered as ‘ordinary’ debts, might have triggered insolvency, or at least pre-insolvency proceedings.

### The Covid-19 Moratorium

The debt enforcement moratorium instrument was already known in Swiss insolvency law (cf. Art. 293 et seqq. DEBA). Though it was recently reformed, it is still a tool rarely used. With the Covid-19 Ordinance on Insolvency Law, the federal government had introduced a new, slightly less complex variant of the moratorium.^17^ With this instrument, SMEs^18^ could apply to the competent court for a temporary deferment of payment in a simplified procedure. The application would be approved under the following (very low-threshold) conditions:

1. The application had to come from an SME (no large companies, no creditor applications);
2. The applicant company had not been over-indebted as of 31 December 2019; and
3. The current financial situation was established and communicated to the court.

Unlike under the ‘ordinary’ (pre-pandemic) enforcement moratorium, it was not necessary to also prove any prospects of restructuring.

Where these conditions were met, the court would grant a Covid-19 deferment of payment for three months (renewable up to a maximum of 6 months). The main purpose of the Covid-19 deferment was to protect the debtor from debt enforcement.^19^ Except for certain privileged claims (in particular, employees’ claims), the debtor was not allowed to pay any claims that arose before the deferment. After its expiry, business activities could continue under the normally applicable rules—including the possibility to enforce debts against the debtor.

This exceptional moratorium was hardly used at all. As a consequence, the Covid-19 moratorium provisions were left to expire in October 2020. Statistics showed that a total of only 25 Covid-19 moratoria (as opposed to about 6000 liquidations) were granted from May to October 2020.^20^ The practical irrelevance of this proceeding was a sobering experience for legislators. Among the lessons that can be learned from this are the following: first, a new form of procedure needs considerable time to take root in the consciousness of practitioners (familiarization) so that it can be effectively implemented; secondly, in a crisis such as the Covid-19 crisis, fiscal measures may be far more effective than insolvency proceedings; finally, where there is no prospect of turnaround, no proceeding can help better than an efficient and swift liquidation.

## Covid-19 Ordinance on Justice and Procedural Law 2020

A further measure taken by the Swiss Federal Council, on 16 April 2020, was aimed at simplifying the service of debt collection documents and the operations of the insolvency offices through its Covid-19 Ordinance on Justice and Procedural Law (‘C19-Justice Ordinance’).^21^ These measures related mainly to operational aspects of the insolvency law regime. For instance, if ‘a first ordinary attempt at service has failed or is impossible or hopeless in the individual case from the outset due to special circumstances’,^22^ service is effected under a simplified procedure not implying mandatory personal service (Art. 7(1) C19-Justice Ordinance).^23^

A further measure that proved to be effective in practice was Art. 9(1) C19-Justice Ordinance referring to public auctions. In deviation from Arts. 125–129 and 257–259 DEBA, the realization of movable assets could also take place through an auction via a publicly accessible online platform. Private commercial platforms such as ‘ricardo.ch’ or ‘ebay.ch’ could be used, as well as debt collection authorities’ own platforms.^24^ In practice, these platforms were already being used, but without a clear legal basis. The introduction of such legal basis encouraged the use of online platforms for auctions during the pandemic.

## Conclusion

The major wave of bankruptcies feared because of the Covid-19 crisis has—so far—not materialized. This is most likely due to emergency financial aid such as short-time work compensation and Covid-19 loans. Other measures such as a suspension of the obligation to file for insolvency and special forms of insolvency proceedings (which both applied for a very limited time) seem to have had a very limited impact, if any.

The initial absolute stay of proceedings proved its worth as an initial stabilization measure—at the same time, it proved a wise decision to limit it to a very short period.^25^ There was no massive disruption of commercial transactions, and the limited time was well used by the debt collection and bankruptcy offices to make operational adaptations.

A big question mark remains with respect to the years to come: the latest numbers indicate that an ‘insolvency wave’ is finally arriving, but in far more modest numbers than initially feared. The main reason for this ‘wave’ may not be the pandemic, but other factors, like the energy crisis or simply a ‘regression to the mean’ effect. However, it is probably too soon to make any claims about this, given the long timeframe for the repayment of Covid-19 loans.

Emergency loans were granted from 26 March to 31 July 2020 based on the Emergency Ordinance on Granting of Credits with Joint and Several Federal Guarantees of 25 March 2020 (‘C19-Solidarity Guarantee Ordinance’).^26^ This was followed, in December 2020, by the Federal Act on Credits with Joint and Several Federal Guarantees as a Result of the Coronavirus (‘C19-Solidarity Guarantee Act’).^27^ The loans were granted for a maximum period of eight years from signature (Art. 3(1) C19-Solidarity Guarantee Act). The repayment period can be extended to ten years in cases of significant hardship (Art. 3(3) C19-Solidarity Guarantee Act). This means that by the end of July 2028, or July 2030 at the latest, the last of the 137,864 emergency loans granted^28^ must be repaid. To date, 30,111 Covid-19 loans have already been repaid,^29^ which corresponds to approx. 22%. The risk is exacerbated by the fact that, as stated under 3.1 (*in finem*), companies that have received Covid-19 loans are currently ‘flying under the radar’ of the financial distress triggers of Art. 725 SCO despite having considerable debts which, if treated as ‘ordinary’ debts, might have triggered insolvency, or at least pre-insolvency proceedings.

All this means that in Switzerland, by around 2030, we can expect a number of companies to be unable to repay their loans and/or to be financially over-indebted as a consequence of the Covid-19 legislation. Whether this will provoke a true insolvency wave will depend on all other economic factors in the years to come (enabling or not repayment of debts) and on the political will to enforce outstanding loans.

## Footnotes

1 Biaggini (2020), p 240.

2 Swiss Federal Department of Finance, Covid-19 credits, https://covid19.easygov.swiss/en/ (accessed 16 September 2022). These measures are discussed in the paper by Luca Jagmetti in this volume.

3 Swiss Confederation, ch.ch, https://www.ch.ch/en/work/short-time-working (accessed 2 September 2022).

4 See https://covid19.easygov.swiss/en/ (accessed 16 September 2022). Granted Covid-19 credits: 137,864. Average amount = CHF 122,796 (ca. EUR 130,000). Granted credit volume = CHF 16,929,104,245 (ca. EUR 17.5 billion). Fully repaid Covid-19 credits = 30,111. This means that approx. 22% of the loans granted have been repaid to date. 101,092 loans are still in progress. Also noteworthy: criminal charges were filed in 2,282 cases on suspicion of abuse. Art. 25(1) C19-Solidarity Guarantee Act: penalty that may be incurred = fine in the amount of CHF 100,000.

5 Swiss Debt Enforcement and Bankruptcy Act, https://www.fedlex.admin.ch/eli/cc/11/529_488_529/de (accessed 16 September 2022).

6 For the calculation, cf. Swiss Federal Court, BGE 108 III 49 consid. 1.

7 Federal Council Decree of 5 August 1914, AS 1914 358; 1914 398. On 13 March 1916, a new legal standstill came into force, until 1920, only for military personnel, AS 1916 83; 1920 587. During the Second World War, the Federal Council renounced declaring any legal standstill.

8 Eichel and Turtschi (2020), p 1010.

9 Covid-19 Ordinance on Insolvency Law, https://www.fedlex.admin.ch/eli/cc/2020/235/de (accessed 16 September 2022).

10 Swiss Federal Council, Press release of 16 April 2020, https://www.admin.ch/gov/de/start/dokumentation/medienmitteilungen/bundesrat.msg-id-78793.html (accessed 2 September 2022).

11 Affolter Marino (2020), p 2; Neuenschwander (2021), p 28. On all measures cited in Sects. 2, 3 and 4, see Molo and Amadò (2020), *passim.*

12 Swiss Code of Obligations, https://www.fedlex.admin.ch/eli/cc/27/317_321_377/en (accessed 15 September 2022).

13 Affolter Marino (2020), p 3.

14 Swiss Confederation, ch.ch, https://www.ch.ch/en/work/short-time-working/ (accessed 2 September 2022).

15 Federal Department of Finance, Covid-19 credits, https://covid19.easygov.swiss/en/ (accessed 2 September 2020).

16 The relevant provision of the emergency ordinance was transposed into Art. 24 of the Federal Covid-19 Solidarity Loans Act (SR 951.26, see https://www.fedlex.admin.ch/eli/cc/2020/982/de, German version (accessed 18 December 2020).

17 Hunkeler et al. (2020), p 314.

18 Public companies and large corporations cannot apply for the Covid-19 deferment, nor can natural persons, ibid.

19 Ibid.

20 Swiss Official Gazette of Commerce, https://www.shab.ch/#!/search/publications?keyword=COVID-19-Stundung (accessed 2 September 2022).

21 Covid-19 Ordinance on Justice and Procedural Law, https://www.fedlex.admin.ch/eli/cc/2020/234/de (accessed 16 September 2022).

22 From the outset, delivery may not appear possible, for example, in certain health or old people’s facilities without sufficient structural protection or with a ban on visitors, or in the case of a business operation that is no longer active.

23 Where the recipient was not served under the ordinary service proceedings (personal notification), he had to be informed in advance (by phone or email) of the imminent mail service. For further details, see Neuenschwander (2021), p 29 et seq.

24 Swiss Federal Department of Justice and Police, *Weisung Nr. 7 vom 16. April 2020 (COVID-19 Wiederaufnahme des Betreibungswesens*), https://www.bj.admin.ch/bj/de/home/wirtschaft/schkg/weisungen.html (accessed 2 September 2022), p 4 et seq.

25 Eichel and Turtschi (2020), p 1021.

26 Fedlex, Emergency Ordinance on Granting of Credits with Joint and Several Federal Guarantees, https://www.fedlex.admin.ch/eli/oc/2020/194/de (accessed 16 September 2022); see also Sect. 1.2 above.

27 Fedlex, Federal Act on Credits with Joint and Several Federal Guarantees as a Result of the Coronavirus, https://www.fedlex.admin.ch/eli/cc/2020/982/de (accessed 16 September 2022).

28 Federal Department of Finance, Covid-19 credits, https://covid19.easygov.swiss/en/ (accessed 16 September 2022).

29 Ibid.
